# 3,3′′,4,4′′-Tetra­meth­oxy-1,1′:4′,1′′-terphen­yl

**DOI:** 10.1107/S1600536811025207

**Published:** 2011-07-02

**Authors:** Law Kung Pui, Wong Woei Hung, Bohari M. Yamin, Mohammad B. Kassim

**Affiliations:** a School of Chemical Sciences & Food Technology, Faculty of Science & Technology, Universiti Kebangsaan Malaysia, 43600 Selangor, Malaysia; b Fuel Cell Institute, Universiti Kebangsaan Malaysia, 43600 Selangor, Malaysia

## Abstract

The title mol­ecule, C_22_H_22_O_4_, is centrosymmetric with an inversion centre located at the centre of the benzene ring. The 3,4-dimeth­oxy­benzene fragment is essentially planar [maximum deviation = 0.400 (2) Å] and twisted relative to the central benzene ring, forming a dihedral angle of 21.25 (7)°. In the crystal, C—H⋯O hydrogen bonds link the mol­ecules into a two-dimensional polymeric structure lying parallel to (100).

## Related literature

For the synthesis, see: Bahadir *et al.* (2003 ▶). For related structures and background references, see: Krummland *et al.* (1997 ▶); Schweigert *et al.* (2001 ▶).
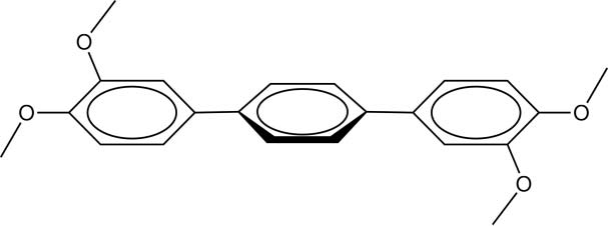


## Experimental

### 

#### Crystal data

C_22_H_22_O_4_
*M* *_r_* = 350.40
Monoclinic, 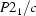

*a* = 13.217 (3) Å
*b* = 8.808 (2) Å
*c* = 8.058 (2) Å
β = 105.476 (4)°
*V* = 904.1 (4) Å^3^
*Z* = 2
Mo *K*α radiation
μ = 0.09 mm^−1^
*T* = 298 K
0.43 × 0.40 × 0.14 mm

#### Data collection

Bruker SMART APEX CCD area-detector diffractometer
Absorption correction: multi-scan (*SADABS*; Bruker, 2000 ▶) *T*_min_ = 0.963, *T*_max_ = 0.988
5114 measured reflections
1866 independent reflections
1166 reflections with *I* > 2σ(*I*)
*R*_int_ = 0.029

#### Refinement

*R*[*F* ^2^ > 2σ(*F* ^2^)] = 0.057
*wR*(*F* ^2^) = 0.160
*S* = 1.04
1866 reflections
120 parameters
H-atom parameters constrained
Δρ_max_ = 0.22 e Å^−3^
Δρ_min_ = −0.13 e Å^−3^

### 

Data collection: *SMART* (Bruker, 2000 ▶); cell refinement: *SAINT* (Bruker, 2000 ▶); data reduction: *SAINT*; program(s) used to solve structure: *SHELXS97* (Sheldrick, 2008 ▶); program(s) used to refine structure: *SHELXL97* (Sheldrick, 2008 ▶); molecular graphics: *SHELXTL* (Sheldrick, 2008 ▶); software used to prepare material for publication: *SHELXTL* and *PLATON* (Spek, 2009 ▶).

## Supplementary Material

Crystal structure: contains datablock(s) I, global. DOI: 10.1107/S1600536811025207/gk2390sup1.cif

Structure factors: contains datablock(s) I. DOI: 10.1107/S1600536811025207/gk2390Isup2.hkl

Supplementary material file. DOI: 10.1107/S1600536811025207/gk2390Isup3.cml

Additional supplementary materials:  crystallographic information; 3D view; checkCIF report

## Figures and Tables

**Table 1:** Hydrogen-bond geometry (Å, °)

*D*—H⋯*A*	*D*—H	H⋯*A*	*D*⋯*A*	*D*—H⋯*A*
C10—H10*A*⋯O1^i^	0.96	2.47	3.331 (3)	149

Symmetry code: (i) 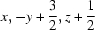.

**Fig. 1.**
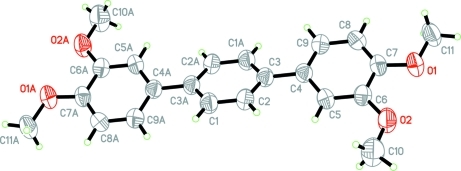
The molecular structure of the title compound with displacement ellipsoids drawn at the 50% probability level. Symmetry code for atoms with the A label: -x, 1-y, 1-z.

**Fig. 2.**
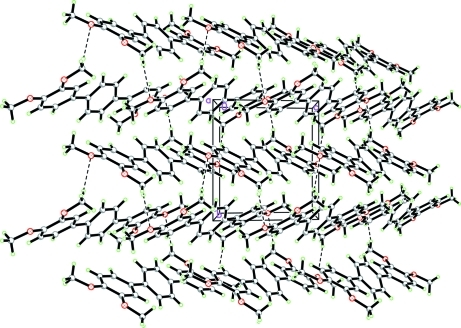
A packing diagram of the title compound viewed down the a-axis showing intermolecular C10—H10A···O1 hydrogen bond.

## supplementary crystallographic information

### Comment

The title compound, is an analog of the previously reported 2,3,8,9-tetramethoxydibenzo[c,e][1,2]dithiin molecules (Krummland *et al.*, 1997). The chemical and biological properties of the related chatecols were also studied by Schweigert *et al.* (2001).

The whole molecule is relatively flat with a maximum deviation from the mean plane at C2 [0.400 (2) Å ]. The central phenyl ring is twisted relative to the 3,4-dimethoxybenzene fragments forming a dihedral angle of 21.25 (7)°. Both methoxy fragments are essentially coplanar with the parent benzene ring with the largest deviation from the mean plane of O1/02/C3/C4/C5/C6/C7/C8/C9/C10/C11 of 0.046 (3) Å for C10.

The crystal structure is stabilized by intermolecular C10—H10A···O1 hydrogen bond linking the molecules into a two dimensional polymeric network parallel to (1 0 0) (Table 1, Fig. 2).

### Experimental

To a 1,4-dibromobenzene (0.236 g, 1 mmol) was added Pd(PPh_3_)_4_ (0.07 g, 0.06 mmol) in dry toluene (6 ml) and stirred for 15 min. Then an aqueous solution of Na_2_CO_3_ (2 ml of 2*M* solution) was added, followed by a 3,4-dimethoxyphenylboronic acid (0.40 g, 2.2 mmol) in EtOH (5 ml). The mixture was refluxed at 95°C for 20 h. The reaction was quenched by adding 30% H_2_O_2_ (0.5 ml) slowly to oxidize the excess 3,4-dimethoxyphenylboronic acid. The reaction mixture was cleaned by NaCl solution (1*M*) and was extracted several times with DCM. The organic residue was washed with 30 ml of water and was dried over CaH_2_. The solvent was removed *in vacuo* and recrystallized from DCM/n-hexane to afford white solids suitable for X-ray single-crystal diffraction (yield: 84%).

### Refinement

All H atoms were postioned geometrically with C—H bond lengths in the range 0.93 - 0.96 Å and refined in the riding model approximation with *U*_iso_(H)=1.2*U*_eq_(C), except for methyl group where *U*_iso_(H)= 1.5*U*_eq_(C).

### Crystal data

**Table:**

C_22_H_22_O_4_	*F*(000) = 372
*M**_r_* = 350.40	*D*_x_ = 1.287 Mg m^−^^3^
Monoclinic, *P*2_1_/*c*	Melting point = 576–578 K
Hall symbol: -P 2ybc	Mo *K*α radiation, λ = 0.71073 Å
*a* = 13.217 (3) Å	Cell parameters from 2137 reflections
*b* = 8.808 (2) Å	θ = 1.6–26.5°
*c* = 8.058 (2) Å	µ = 0.09 mm^−^^1^
β = 105.476 (4)°	*T* = 298 K
*V* = 904.1 (4) Å^3^	Block, colourless
*Z* = 2	0.43 × 0.40 × 0.14 mm

### Data collection

**Table:**

Bruker SMART APEX CCD area-detector diffractometer	1866 independent reflections
Radiation source: fine-focus sealed tube	1166 reflections with *I* > 2σ(*I*)
graphite	*R*_int_ = 0.029
ω scans	θ_max_ = 26.5°, θ_min_ = 1.6°
Absorption correction: multi-scan (*SADABS*; Bruker, 2000)	*h* = −16→15
*T*_min_ = 0.963, *T*_max_ = 0.988	*k* = −6→11
5114 measured reflections	*l* = −10→10

### Refinement

**Table:**

Refinement on *F*^2^	Primary atom site location: structure-invariant direct methods
Least-squares matrix: full	Secondary atom site location: difference Fourier map
*R*[*F*^2^ > 2σ(*F*^2^)] = 0.057	Hydrogen site location: inferred from neighbouring sites
*wR*(*F*^2^) = 0.160	H-atom parameters constrained
*S* = 1.04	*w* = 1/[σ^2^(*F*_o_^2^) + (0.0871*P*)^2^] where *P* = (*F*_o_^2^ + 2*F*_c_^2^)/3
1866 reflections	(Δ/σ)_max_ < 0.001
120 parameters	Δρ_max_ = 0.22 e Å^−^^3^
0 restraints	Δρ_min_ = −0.13 e Å^−^^3^

### Special details

**Table:**

Geometry. All e.s.d.'s (except the e.s.d. in the dihedral angle between two l.s. planes) are estimated using the full covariance matrix. The cell e.s.d.'s are taken into account individually in the estimation of e.s.d.'s in distances, angles and torsion angles; correlations between e.s.d.'s in cell parameters are only used when they are defined by crystal symmetry. An approximate (isotropic) treatment of cell e.s.d.'s is used for estimating e.s.d.'s involving l.s. planes.
Refinement. Refinement of *F*^2^ against ALL reflections. The weighted *R*-factor *wR* and goodness of fit *S* are based on *F*^2^, conventional *R*-factors *R* are based on *F*, with *F* set to zero for negative *F*^2^. The threshold expression of *F*^2^ > σ(*F*^2^) is used only for calculating *R*-factors(gt) *etc*. and is not relevant to the choice of reflections for refinement. *R*-factors based on *F*^2^ are statistically about twice as large as those based on *F*, and *R*- factors based on ALL data will be even larger.

### Fractional atomic coordinates and isotropic or equivalent isotropic displacement parameters (Å^2^)

**Table:**

	*x*	*y*	*z*	*U*_iso_*/*U*_eq_	
O1	0.35830 (11)	0.46473 (15)	−0.01311 (17)	0.0655 (5)	
O2	0.41248 (11)	0.60399 (17)	0.27724 (18)	0.0718 (5)	
C1	0.01164 (14)	0.6080 (2)	0.6254 (2)	0.0588 (5)	
H1	0.0205	0.6821	0.7103	0.071*	
C2	0.07975 (14)	0.6046 (2)	0.5231 (2)	0.0576 (5)	
H2	0.1332	0.6763	0.5405	0.069*	
C3	0.07065 (14)	0.4961 (2)	0.3938 (2)	0.0521 (5)	
C4	0.14417 (14)	0.4894 (2)	0.2832 (2)	0.0517 (5)	
C5	0.24440 (15)	0.5542 (2)	0.3353 (2)	0.0523 (5)	
H5	0.2648	0.6042	0.4406	0.063*	
C6	0.31329 (14)	0.5458 (2)	0.2345 (2)	0.0517 (5)	
C7	0.28368 (15)	0.4708 (2)	0.0755 (2)	0.0525 (5)	
C8	0.18531 (16)	0.4092 (2)	0.0216 (2)	0.0626 (6)	
H8	0.1644	0.3607	−0.0845	0.075*	
C9	0.11677 (15)	0.4189 (2)	0.1246 (2)	0.0630 (6)	
H9	0.0502	0.3765	0.0856	0.076*	
C10	0.44505 (19)	0.6888 (3)	0.4319 (3)	0.0965 (9)	
H10A	0.3951	0.7680	0.4316	0.145*	
H10B	0.5127	0.7329	0.4402	0.145*	
H10C	0.4495	0.6230	0.5285	0.145*	
C11	0.33034 (18)	0.3959 (3)	−0.1786 (2)	0.0817 (7)	
H11A	0.3120	0.2916	−0.1680	0.123*	
H11B	0.3887	0.4012	−0.2282	0.123*	
H11C	0.2714	0.4485	−0.2514	0.123*	

### Atomic displacement parameters (Å^2^)

**Table:**

	*U*^11^	*U*^22^	*U*^33^	*U*^12^	*U*^13^	*U*^23^
O1	0.0763 (10)	0.0667 (10)	0.0657 (9)	−0.0023 (7)	0.0402 (8)	−0.0057 (7)
O2	0.0695 (9)	0.0792 (10)	0.0783 (10)	−0.0193 (7)	0.0396 (8)	−0.0218 (8)
C1	0.0577 (12)	0.0623 (13)	0.0608 (12)	0.0026 (10)	0.0234 (10)	−0.0068 (9)
C2	0.0527 (11)	0.0603 (13)	0.0644 (13)	0.0003 (9)	0.0239 (10)	−0.0030 (10)
C3	0.0516 (11)	0.0544 (12)	0.0532 (12)	0.0090 (9)	0.0188 (9)	0.0045 (9)
C4	0.0508 (11)	0.0544 (11)	0.0521 (11)	0.0085 (9)	0.0177 (9)	0.0044 (9)
C5	0.0601 (12)	0.0500 (11)	0.0521 (11)	0.0028 (9)	0.0242 (9)	−0.0016 (8)
C6	0.0537 (11)	0.0449 (11)	0.0604 (12)	−0.0003 (8)	0.0221 (9)	0.0026 (9)
C7	0.0602 (12)	0.0492 (11)	0.0554 (12)	0.0070 (9)	0.0278 (10)	0.0072 (9)
C8	0.0647 (13)	0.0762 (14)	0.0487 (11)	0.0024 (11)	0.0181 (10)	−0.0067 (10)
C9	0.0533 (12)	0.0790 (15)	0.0593 (13)	−0.0023 (10)	0.0196 (10)	−0.0043 (10)
C10	0.0924 (17)	0.093 (2)	0.116 (2)	−0.0364 (14)	0.0489 (16)	−0.0488 (16)
C11	0.0906 (17)	0.1048 (19)	0.0606 (14)	0.0140 (14)	0.0388 (13)	−0.0016 (12)

### Geometric parameters (Å, °)

**Table:**

O1—C7	1.364 (2)	C5—C6	1.374 (2)
O1—C11	1.421 (2)	C5—H5	0.9300
O2—C6	1.364 (2)	C6—C7	1.401 (3)
O2—C10	1.418 (2)	C7—C8	1.368 (3)
C1—C2	1.373 (3)	C8—C9	1.385 (3)
C1—C3^i^	1.400 (3)	C8—H8	0.9300
C1—H1	0.9300	C9—H9	0.9300
C2—C3	1.395 (3)	C10—H10A	0.9600
C2—H2	0.9300	C10—H10B	0.9600
C3—C1^i^	1.400 (3)	C10—H10C	0.9600
C3—C4	1.484 (3)	C11—H11A	0.9600
C4—C9	1.380 (3)	C11—H11B	0.9600
C4—C5	1.400 (3)	C11—H11C	0.9600
			
C7—O1—C11	117.68 (15)	O1—C7—C6	115.63 (17)
C6—O2—C10	117.78 (15)	C8—C7—C6	119.13 (17)
C2—C1—C3^i^	122.27 (18)	C7—C8—C9	120.26 (18)
C2—C1—H1	118.9	C7—C8—H8	119.9
C3^i^—C1—H1	118.9	C9—C8—H8	119.9
C1—C2—C3	121.59 (18)	C4—C9—C8	122.05 (19)
C1—C2—H2	119.2	C4—C9—H9	119.0
C3—C2—H2	119.2	C8—C9—H9	119.0
C2—C3—C1^i^	116.14 (17)	O2—C10—H10A	109.5
C2—C3—C4	122.42 (18)	O2—C10—H10B	109.5
C1^i^—C3—C4	121.43 (17)	H10A—C10—H10B	109.5
C9—C4—C5	117.06 (17)	O2—C10—H10C	109.5
C9—C4—C3	121.46 (17)	H10A—C10—H10C	109.5
C5—C4—C3	121.48 (17)	H10B—C10—H10C	109.5
C6—C5—C4	121.58 (18)	O1—C11—H11A	109.5
C6—C5—H5	119.2	O1—C11—H11B	109.5
C4—C5—H5	119.2	H11A—C11—H11B	109.5
O2—C6—C5	125.06 (17)	O1—C11—H11C	109.5
O2—C6—C7	115.03 (16)	H11A—C11—H11C	109.5
C5—C6—C7	119.90 (17)	H11B—C11—H11C	109.5
O1—C7—C8	125.24 (18)		

Symmetry codes: (i) −*x*, −*y*+1, −*z*+1.

### Hydrogen-bond geometry (Å, °)

**Table:**

*D*—H···*A*	*D*—H	H···*A*	*D*···*A*	*D*—H···*A*
C10—H10A···O1^ii^	0.96	2.47	3.331 (3)	149

Symmetry codes: (ii) *x*, −*y*+3/2, *z*+1/2.
